# Differential lexical predictors of reading comprehension in fourth graders

**DOI:** 10.1007/s11145-016-9686-0

**Published:** 2016-09-03

**Authors:** Nicole M. Swart, Marloes M. L. Muijselaar, Esther G. Steenbeek-Planting, Mienke Droop, Peter F. de Jong, L. Verhoeven

**Affiliations:** 1 Behavioural Science Institute, Radboud University, P.O. Box 9104, 6500 HE Nijmegen, The Netherlands; 2 Research Institute of Child Development and Education, University of Amsterdam, Nieuwe Prinsengracht 127, 1018 WS Amsterdam, The Netherlands

**Keywords:** Reading comprehension, Vocabulary, Mental lexicon, Fourth grade

## Abstract

The mental lexicon plays a central role in reading comprehension (Perfetti & Stafura, 2014). It encompasses the number of lexical entries in spoken and written language (vocabulary breadth), the semantic quality of these entries (vocabulary depth), and the connection strength between lexical representations (semantic relatedness); as such, it serves as an output for the decoding process and as an input for comprehension processes. Although different aspects of the lexicon can be distinguished, research on the role of the mental lexicon in reading comprehension often does not take these individual aspects of the lexicon into account. The current study used a multicomponent approach to examine whether measures of spoken and written vocabulary breadth, vocabulary depth, and semantic relatedness were differentially predictive of individual differences in reading comprehension skills in fourth-grade students. The results indicated that, in addition to nonverbal reasoning, short-term memory, and word decoding, the four measures of lexical quality substantially added (30 %) to the proportion of explained variance of reading comprehension (adding up to a total proportion of 65 %). Moreover, each individual measure of lexical quality added significantly to the prediction of reading comprehension after all other measures were taken into account, with written lexical breadth and lexical depth showing the greatest increase in explained variance. It can thus be concluded that multiple components of lexical quality play a role in children’s reading comprehension.

## Introduction

Reading comprehension has been defined as the process of extracting and constructing meaning from written text. The reader has to create a mental representation of the text, or in other words, a situation model integrating text information with the reader’s prior knowledge (Kintsch, 1988, 2012; Van Dijk & Kintsch, 1983). In creating this representation, different higher- and lower-order processes (e.g., word decoding, inference making, meaning retrieval, monitoring) play a role (e.g., Nation, 2005; Ouellette & Beers, 2010; Perfetti, Landi, & Oakhill, 2005; Van den Broek, 1994). A great deal of research on reading comprehension has focused on listening comprehension and word decoding in explaining individual differences (Cutting & Scarborough, 2006; Gough & Tunmer, 1986; Hoover & Gough, 1990; Tilstra, McMaster, Van den Broek, Kendeou, & Rapp, 2009), leaving the impact of lexical or vocabulary processes underexplored. Therefore, the present study examines the role of differential lexical predictors of fourth graders’ reading comprehension.

To understand the complex process of reading comprehension, a general framework highlighting differential components is necessary. Perfetti and Stafura’s (2014) Reading Systems Framework (RSF) provides such a framework in which the mental lexicon plays a central role, being a connection point between word identification and text comprehension processes. When reading a text, the lexicon serves as an output source for the word identification process in which orthographic and phonological pieces of information are combined into single words. In addition, information from the lexicon also serves as an input source for comprehension-related processes in which single words are combined into comprehensive sentences and passages. Consequently, problems with the mental lexicon often result in comprehension difficulties. Although numerous studies have shown that children with more semantic knowledge are better able to understand written texts (e.g., Verhoeven & Van Leeuwe, 2008; Verhoeven, Van Leeuwe, & Vermeer, 2011), little is known about the relationship between specific dimensions of semantic knowledge and text comprehension. The current study used a multicomponent approach to examine how the mental lexicon modulates reading comprehension in fourth graders. Different aspects of the mental lexicon were examined in order to explain individual differences in reading comprehension.

The RSF distinguishes three sources of knowledge: linguistic knowledge, orthographic knowledge, and general background knowledge (Perfetti & Stafura, 2014). Different processes enable us to use and combine these sources of knowledge in order to understand written texts. Word decoding and word identification processes are necessary to make sense of the written units. Meaning retrieval, sentence building, inference, and monitoring processes, in turn, are required to combine the single words into a meaningful passage. The RSF is consistent with Hagoort’s (2005, 2007) Memory, Unification, and Control (MUC) model of language, a neurobiological model of language processing. Based on neurological evidence, three functional components can be distinguished (for a review, see Hagoort, 2013). First, the memory component is involved in retrieving information from long-term memory. By reading every single word, the knowledge of word meanings, connections to other words, and prior knowledge are retrieved from long-term memory. After retrieving this information, the unification component facilitates processes resulting in the combination of these pieces of information into larger units, such as sentences and passages. Finally, the control component ensures that the intended actions are carried out. As a case in point, executive control processes are needed to read multiple sources of text or to relate different parts of a text to one another.

As previously mentioned, the mental lexicon plays a central role in reading comprehension, serving as both an input and an output source (Perfetti & Stafura, 2014). It can be defined as the place where word representations are stored in long-term memory. Each representation corresponds to a word known to a more or lesser extent, resulting in individual vocabularies. Two dimensions are often distinguished: vocabulary breadth and vocabulary depth (e.g., Cain, 2010; Ouellette, 2006; Vermeer, 2001). Vocabulary breadth refers to the number or quantity of spoken and written word representations stored in the lexicon. Vocabulary depth refers to the quality of the representations stored in the lexicon. Previous research has indicated that skilled comprehenders differ from less skilled comprehenders in both quality and quantity of these lexical representations (Braze, Tabor, Shankweiler, & Mencl, 2007; Cain & Oakhill, 2014; Kendeou, Savage, & Van den Broek, 2009; Ouellette, 2006; Ouellette & Beers, 2010; Ricketts, Nation, & Bishop, 2007; Tannenbaum, Torgeson, & Wagner, 2006; Tilstra et al., 2009; Verhoeven & Van Leeuwe, 2008). Skilled comprehenders tend to know more words, and their knowledge of these words is more extensive compared to that of less skilled comprehenders, demonstrating the importance of a well-developed lexicon in relation to reading comprehension skills.

Whereas the RSF is a general framework bringing together comprehension-related processes, other theories, such as the Triangle model (Plaut, McClelland, Seidenberg, & Patterson, 1996) and the Lexical Quality Hypothesis (LQH; Perfetti & Hart, 2002), describe in more detail the relationship between word representations stored in the mental lexicon and reading comprehension. According to these models, each word representation or entry in the mental lexicon consists of three constituents or chunks of information: orthographic information, phonological information, and semantic information. Individual differences in reading comprehension can be deduced from individual differences in the quantity and quality of these lexical representations (Perfetti, 2007). The quality of a representation is high when orthographic, phonological, and semantic knowledge are well developed—in other words, when someone knows how to spell and pronounce the word and knows what its meaning is. Readers with a rich lexicon with many high quality representations comprehend written texts better than those with fewer representations that are usually of lower quality. In addition to the quality of the individual constituents, connection strength between the constituents is predictive of reading comprehension skill. In a factor analysis with various lexical quality tasks, Perfetti and Hart (2002) found that, for skilled adult readers, two factors could be distinguished: one factor for the orthographic and phonological tasks and one factor for the semantic tasks. Meanwhile, for less skilled adult readers, three factors could be extracted, one for each aspect of lexical quality. These results indicate that, in less skilled adult readers, the constituents are not tightly bound together, which might result in reading comprehension difficulties. Research involving primary school students has also demonstrated the importance of well-developed semantic knowledge in reading comprehension. Richter, Isberner, Naumann, and Neeb (2013) argued that grade-level differences in reading comprehension can be fully explained by individual differences in lexical quality. The authors concluded that deficits in the semantic constituent (not knowing the meaning of a word) can result in reading comprehension difficulties, even when orthographic and phonological constituents are of high quality.

The organization of the mental lexicon can be compared to a web of interconnected elements (e.g., Bock & Levelt, 1994). Representations can be connected based on, for example, semantic information. According to spreading activation models, the activation of one representation leads to the parallel activation of connected representations (Collins & Loftus, 1975). These connections can be based on different types of relationships and include antonyms, synonyms, super- and subordinate relations, category members, and functional relationships. In developing a strong network, connections start out weak, and parallel activation is not always strong enough to activate related representations. However, when connections are more often encountered in both written and spoken language, connections become stronger and the parallel activation of related representations more often succeeds. Evidence from priming studies suggests that strong comprehenders show signs of stronger word connections compared to poor comprehenders (e.g., Betjemann & Keenan, 2008; Cronin, 2002; Nation & Snowling, 1999). Therefore, having strong connections between word representations might also aid the comprehension process.

To summarize, the mental lexicon plays a central role in reading comprehension processes. Comprehension skills are related to both the quantity and quality of the representations stored in the lexicon. Previous research has shown that, to become skilled comprehenders, readers need to develop a strong lexicon, with many high quality word representations and strong connections between these representations. However, most studies on vocabulary and reading comprehension have included only one aspect of the lexicon in their design, under-exposing the multidimensional nature of the mental lexicon. Therefore, the current paper attempts to examine the relationship between different aspects of the lexicon and reading comprehension skills of children in the fourth grade of primary school. To this end, we used a multicomponent approach to measure three aspects of the lexicon: breadth, depth, and strength of connections between words. The present study provides more knowledge about the role of the mental lexicon in reading comprehension. Our research question was: How are individual differences in differential components of the mental lexicon related to individual differences in reading comprehension skills?

Previous research has indicated that a connection exists between vocabulary breadth (e.g., Verhoeven et al., 2011), vocabulary depth (e.g., Ricketts et al., 2007), and connection strength (Betjemann & Keenan, 2008), on the one hand, and reading comprehension, on the other hand (e.g., Cronin, 2002). Thus, the current study measured these three dimensions of the mental lexicon. Size of the lexicon (or vocabulary breadth) was measured using two tests: an oral and a written vocabulary breadth test. The quality of semantic knowledge (or vocabulary depth) was measured using a word definition task. Finally, connection strength between representations was measured using a word association task. To be able to examine the individual contributions of these lexical predictors, we controlled for some other well-known predictors of reading comprehension—namely, decoding (e.g., Verhoeven & Van Leeuwe, 2008), short-term memory (e.g., Nation, Adams, Bowyer-Crane, & Snowling, 1999), and general reasoning (e.g., Fuchs et al., 2012). We hypothesized that quantity and quality of semantic knowledge and the strength of the word connections were positively related to reading comprehension skills, in that children who know more words, who have deeper word knowledge, and stronger word connections would also have better developed reading comprehension skills. Due to the fact that the reading comprehension and written vocabulary breadth tasks both depend on word decoding skills, it was expected that these two would have the strongest relationship.

## Methods

### Participants

In total, 292 children (147 boys, 145 girls) from 11 different primary schools were tested at the start of the fourth grade (*M*_age_ = 9 years, 7 months; *SD*_age_ = 5.73 months). Schools were located in both urban and suburban regions of the Netherlands. Prior to testing, informed consent was obtained from the parents of the participating children. Of the 292 children, 258 children spoke Dutch with both parents. The remaining 44 children indicated that they spoke Dutch and another language at home. Participants in the current study took part in a larger longitudinal study on reading comprehension development.

### Materials

Thirteen tests were used to measure reading comprehension skills, vocabulary, decoding skills, short-term memory, and nonverbal cognitive reasoning.

#### Reading comprehension

Reading comprehension is a complex process, and different tests might vary in the underlying skills they assess (e.g., Keenan, Betjemann, & Olson, 2008; Kintsch, 2012; Nation & Snowling, 1997). Therefore, four tests, differing in passage length, text type, and question type, were used to measure reading comprehension skills. By including diverse tasks, we aimed to capture the complex nature of reading comprehension skills. Test results were combined in order to get a single component score reflecting comprehension skills.

##### Short passage comprehension

A standardized test for students in the final grades of primary school was used to measure short passage reading comprehension skills (*Begrijpend lezen 678* [Reading comprehension grade 4, 5, 6]; Aarnoutse & Kapinga, 2005). The test consisted of three narrative and four expository passages, containing 123–288 words (mean: 192 words per text). For each passage, students had to answer six or seven questions, resulting in 44 questions total (22 multiple choice with four options and 22 true/false). Questions related to the knowledge and strategies necessary to determine the meaning of words (e.g., what is the meaning of the word?), single sentences (e.g., is this sentence true or false with respect to what you have read in the text?), complete passages (e.g., what is the message the author wants to convey?), and relationships between sentences (e.g., who is referred to by the word “she”?). For each correct answer, students received one point, bringing the maximum score to 44 points. The developers of the test reported a Cronbach’s alpha of .86 as a measure of reliability.

##### Cito reading comprehension scale

Participating schools provided the researchers with results from a standardized test battery used by primary schools throughout the Netherlands to monitor reading comprehension development from first to sixth grades. Results from the test administered halfway through third grade were used in the present study (Cito, 2007). The test is divided over two parts and is partly adaptive to the student’s reading level. Each part consists of a mix of short and medium-long passages (131–634 words; mean number of words per text is 268) and a number of multiple choice questions per text. Both narrative and expository passages were included. Two types of questions were present: passage-based questions, which asked children literal questions about the content of the passages and whose answers could be derived from information literally stated in the passage, and questions requiring children to combine information explicitly stated in the passage with information not stated explicitly in the passage. Standardized scores were used. As indicated in the testing manual, the Accuracy of measurement (measure of test reliability) was >.89.

##### Narrative text reading comprehension

Narrative text comprehension was measured with one text from the Progress in International Reading Literacy Studies (PIRLS) Reading Literacy Test—2011 (Mullis, Martin, Kennedy, Trong, & Sainsbury, 2009): *De vijandentaart* [Enemy Pie]. The narrative text was relatively long and consisted of 832 words. In total, students answered 16 questions: seven multiple choice questions with four options and nine open-ended questions. Questions were literal (to assess understanding of information explicitly stated in the text), inferential (to assess inference skills), or evaluative (to examine how well students were able to evaluate information stated in the text). For the open-ended questions, students received a maximum of one, two, or three points, depending on the difficulty of the question and the quality of the answer. One-point questions required students to answer in single words or a short sentence. For questions worth two or three points, students had to answer in multiple sentences. The maximum test score was 19 points. Responses were scored by four trained research assistants based solely on the completeness of the answer, not on any spelling or grammatical mistakes. Training provided to the scorers included discussing the correct answers provided in the original scoring guide together with some example answers with the first or second author. After training, each research assistant scored the answers of 10 students participating in the study. A high degree of agreement was reached (ICC = .97). Disagreements were resolved by the first and second authors and discussed with the research assistants to reach full agreement. The Cronbach’s alpha, indicating test reliability, was .77.

##### Expository text reading comprehension

Expository text comprehension was measured using a different text from the PIRLS tests (Mullis et al., 2009)—namely, *Het mysterie van de reuzentand* [The mystery of the giant tooth]. The text was relatively long (884 words). Students answered 14 questions, again with one point for each correct multiple choice question (eight in total) and one, two, or three points for every open-ended question (six in total). Questions tapped into literal understanding of the text, inferential abilities, and evaluative skills. Responses to the open-ended questions were scored by the same research assistants who scored the open-ended questions for the narrative reading comprehension test. The same training procedure used for scoring the narrative text was followed. Inter-rater reliability was established by calculating the interclass correlation. Again, a high degree of agreement was reached (ICC = .90), and disagreements were resolved by the first and second authors and discussed with the research assistants. The Cronbach’s alpha was .75.

#### Vocabulary breadth

Two tests were used to assess vocabulary breadth: an oral and a written test. The choice for both an oral and written task originated from the fact that written tests depend on decoding skills whereas oral tests do not.

##### Oral vocabulary breadth

The present study used an adapted version of the Peabody Picture Vocabulary Test (PPVT; Dunn, Dunn, & Schlichting, 2005) so that the test could be administered to a group. A booklet presented the items from sets eight to thirteen of the original PPVT (72 items total). For each item, the four answer options (pictures) were printed next to each other. Students were orally presented with the target word by the experimenter. Students had to underline the picture in their booklet that best matched the target word. The test score was equal to the number of correct items. The Cronbach’s alpha, indicating test reliability for the self-adapted versions, was .69.

##### Written vocabulary breadth

The reading vocabulary subtest from the *Taaltoets Allochtone Kinderen* [Language Test for Foreign Children] (Verhoeven & Vermeer, 1986) was used to measure the vocabulary knowledge of written words. Although the test name might imply differently, the task was used for all children. The tasks consisted of 50 multiple-choice items. In each item, students read a sentence and had to indicate what the underlined word meant by choosing one of the four options listed below the sentence. Test scores were equal to the number of items correct. The Cronbach’s alpha was .82.

#### Vocabulary depth

Measures of vocabulary depth generally ask children to give word definitions. To gain insights into the vocabulary depth, students completed the vocabulary subtest of the Dutch version of the Wechsler Intelligence Scale for Children, third edition (WISC-III NL; Kort et al., 2005). The subtest consisted of 35 items for which children had to give a definition. Answers were scored using the original WISC-III scoring guide. The test score was the total number of points received. The Cronbach’s alpha was .82.

#### Connection strength

Word association tasks can be used to examine lexical-semantic organization (e.g., Aitchinson, 2012; Entwisle, 1966). In the current study, the students were asked to write down at least two and no more than five associations for each of the 20 target nouns (e.g., dog, finger, and spoon). Selected target nouns all had a high frequency to ensure that all students knew the words. For each association-target pair, a score for the association strength was calculated. These scores were based on the existing word association norm list of De Deyne, Navarro, and Storms (2013). In this norm list, 100 students’ associations for 1424 Dutch words were presented. For each association-target pair in the current study, it was calculated how often it occurred in De Deyne and colleagues’ norm list. This procedure resulted in a percentage score for each association-target pair, with a maximum of 100 (20 targets × 5 associations) percentage scores. These percentage scores reflected the association strength between the association and the target words, with high scores reflecting strong associations and low scores reflecting low association. For each student, the mean of all these percentage scores was calculated and used as a measure of the strength of the semantic network. The Cronbach’s alpha was .63.

#### Decoding skills

To measure decoding skills, word and non-word decoding tasks were administered. Test results of both word and non-word reading tasks were combined in order to get one component score reflecting decoding skills.

##### Word reading

The *Een Minuut Test (EMT)* [1 Min Test] (Brus & Voeten, 1999) was used to measure word decoding. The test consisted of a card with 116 words increasing in length, starting with simple CVC words and finishing with multi-syllable words. Students were instructed to read the words as quickly and accurately as possible, resulting in a combined score of both reading rate and reading accuracy. The score on this test was the number of words read correctly within 1 min. The Cronbach’s alpha, as established by the developers of the test, was .89.

##### Non-word reading

To measure non-word decoding skills, *De Klepel* (Van den Bos, Lutje Spelberg, Scheepstra, & de Vries, 1994) was administered. The test consisted of a card with 116 non-words increasing in length and difficulty (equal to the word reading task). Students were instructed to read the non-words as quickly and accurately as possible. The score on this test was the number of words read correctly within 2 min. The Cronbach’s alpha was .93.

#### Short-term memory

Students completed both digit span and word span tasks. In both span tasks, children were orally presented with a sequence of units and were asked to remember the sequence and reproduce it. For the digit span task, the units to be remembered were single-digit units; for the word span task, the units to be remembered were simple high frequency one-syllable words (e.g., ball, bike, door). Both tasks started out relatively easy, with only two units that had to be remembered. For both tasks, difficulty increased gradually to nine units. The digits and words were read to the children by the experimenter at a pace of one unit per second, with a pause of one second between each unit. For each level of difficulty, children received three attempts, and testing was terminated when all three attempts for one difficulty level were incorrect. The number of correctly recalled sequences comprised the scores for both tasks. Test results of both tasks were combined in order to get one component score reflecting short-term memory.

#### Nonverbal cognitive reasoning

To measure nonverbal cognitive reasoning, children completed the Raven Standard Progressive Matrices (SPM; Raven, 1960). Children were presented with 60 visual patterns divided over five sets of increasing difficulty. In each pattern, a piece of the puzzle was missing, and children were asked to indicate which of the six (for the first two sets) or eight (for sets three, four, and five) presented puzzle pieces would complete the pattern. Test scores were the sum of the number of correct answers. The Cronbach’s alpha was .89.

### Procedure

All tests, except the Cito reading comprehension scale, were administered by the researchers at the start of fourth grade. The vocabulary subtest of the WISC-III NL, the word reading task, the non-word reading task, and the short-term memory tasks were completed during two individual test sessions, each taking approximately 20 min. Individual testing took place in a quiet room in the school. All other tests were administered group-wise during three separate sessions. During the first session, students completed both PIRLS texts. This session took approximately 90 min, with a short break between the two tests. In the second session, students completed the short passage reading comprehension task, the word association task, and the adapted version of the PPVT. This second session took approximately 2 h, with short breaks between the tests. On a third morning, students completed the Raven SPM. The Cito reading comprehension scale test was administered in class by the teacher halfway through third grade.

### Data analyses

A principal component analysis (PCA) was conducted on the four reading comprehension tests to examine the factor structure. The PCA was conducted with R (R Core Team, 2015), an open source statistical program, using the principal function from the psych package (Revelle, 2014). The Kaiser–Meyer–Olkin measure of sampling adequacy, as an indicator of appropriateness of performing a factor analysis, was great (KMO = .84), indicating that performing a factor analysis should yield a distinct and reliable factor (Field, Miles, & Field, 2012). Bartlett’s test of sphericity indicated that correlations were large enough to perform a PCA (*X* ^2^ (6) = 681.83, *p* < .001). The PCA, using Kaiser’s criterion to retain only factors with an eigenvalue >1, yielded a one-factor solution that explained 76 % of the variance. All four reading comprehension tests loaded highly on the reading comprehension skills component (eigenvalue = 3.05). The factor loadings were as follows: short passage comprehension = 0.85; Cito reading comprehension scale = 0.89; narrative text reading comprehension = 0.88; and expository text reading comprehension = 0.88. Standardized test scores were combined to generate one composite score for reading comprehension. In addition, the same procedure was followed with the word reading and non-word reading task to create a composite score for decoding skills as well as with the two short-term memory tasks to create a composite score for short-term memory skills.

To assess the unique impact of the different dimensions of vocabulary on fourth graders’ reading comprehension skills, a complete regression model including all predictors was compared to four reduced regression models. In each reduced model, one of the four predictors of semantic quality was left out to calculate variance explained in predicting reading comprehension by each predictor. In the first reduced model, the measure of written vocabulary breadth was left out; in the second reduced model, the measure of oral vocabulary breadth was left out; in the third reduced model, the measure of vocabulary depth was left out; and in the fourth reduced model, the measure of semantic relatedness was left out.

## Results

Descriptive statistics and correlations among the 13 measures are presented in Table 1. Strong correlations were found between the four reading comprehension measures (all *r*’s > .66, *p* < .001), between word and non-word reading tasks (*r* = .85, *p* < .001), and between the two measures of short-term memory (*r* = .55, *p* < .001). As previously described, composite scores were calculated for reading comprehension skills, decoding skills, and short-term memory skills. Correlations between the vocabulary tasks were medium to high.

**Table 1:** Summary of the correlations, means, and standard deviations (n = 292)

Variable	1 NI	2 DS	3 WS	4 WR	5 NWR	6 VBO	7 VBW	8 VD	9 CS	10 SPRC	11 Cito RC	12 NRC	13 ERC
1. NI	–												
2. DS	.200**	–											
3. WS	.151*	.553***	–										
4. WR	.147*	.250***	.214***	–									
5. NWR	.170**	.285***	.207***	.849***	–								
6. VBO	.269***	.109	.090	.245***	.196**	–							
7. VBW	.323***	.183**	.133*	.357***	.280***	.591***	–						
8. VD	.207***	.135*	.152**	.213***	.157**	.492***	.526***	–					
9. CS	.170**	.041	.124*	.077	.013	.230***	.263***	.309***	–				
10. SPRC	.397***	.230***	.194**	.313***	.299***	.487***	.652***	.476***	.304***	–			
11. Cito RC	.428***	.259***	.239***	.364***	.310***	.459***	.593***	.478***	.324***	.675***	–		
12. NRC	.411***	.263***	.198**	.325***	.288***	.471***	.609***	.539***	.350***	.637***	.728***	–	
13. ERC	.479***	.280***	.212***	.325***	.330***	.460***	.578***	.422***	.274***	.661***	.695***	.700***	–
M	42.24	11.08	9.72	60.85	51.66	35.10	27.28	31.46	324.05	26.32	26.91	11.12	8.23
SD	5.75	2.04	1.65	13.71	16.43	6.03	7.12	5.94	68.72	6.00	14.08	4.02	3.48

*RC* reading comprehension, *NI* nonverbal intelligence, *DS* digit span, *WS* word span, *WR* word reading, *NWR* non-word reading, *VBO* vocabulary breadth oral, *VBW* vocabulary breadth written, *VD* vocabulary depth, *CS* connection strength, *SPRC* short passage reading comprehension, *Cito RC* Cito reading comprehension scale, *NRC* narrative reading comprehension, *ERC* expository reading comprehension

* *p* < .05; ** *p* < .01; *** *p* < .001

### Predictors of reading comprehension

A hierarchical regression analysis (HRA), using the lm function in R (R Core Team, 2015), was carried out to gain insight into the impact of vocabulary on reading comprehension skills. Table 2 shows the results of the HRA. In the first step, two general cognitive control measures (nonverbal reasoning and short-term memory) were included to ensure that subsequent effects were not due to differences in these measures: *F*(2,289) = 58.16, *p* < .001, *R*_*adj*_^2^ = .28. In the second step, decoding was included to control for the well-established effect of decoding skills on reading comprehension skills: *F*(3,288) = 52.27, *p* < .001, ∆*R*_*adj*_^2^ = .06. In the third step, all the vocabulary variables were included: *F*(7,384) = 76.64, *p* < .001, ∆*R*_*adj*_^2^ = .30. The results of the final step indicated significant effects for all predictors (nonverbal reasoning, short-term memory, decoding, written vocabulary breadth, oral vocabulary breadth, vocabulary depth, and connection strength); higher scores on each of these predictors resulted in higher scores on the reading comprehension tests. Relatively large standardized coefficients were found for nonverbal reasoning (*β* = .24) and reading vocabulary (*β* = .38), indicating stronger effects for these variables compared to the other predictors. All four measures of vocabulary were significantly related to reading comprehension skills (all *p*’s < .04), with written vocabulary breadth having the largest standardized coefficient (*β* = .38) and oral vocabulary breadth the smallest (*β* = .10). Additional analyses were conducted to examine the relative individual contribution of each of these vocabulary tasks. Together, all predictors explained 65 % of the variance in reading comprehension skill.

**Table 2:** Hierarchical regression analysis predicting reading comprehension skills from nonverbal reasoning, short-term memory, decoding, vocabulary breadth, vocabulary depth, and connection strength

Predictor	∆*R* ^2^	*B*	*SE B*	β
Step 1	.282***			
Constant		−3.10	0.74	–
Nonverbal reasoning		1.56	0.18	0.45***
Short-term memory		0.28	0.07	0.22***
Step 2	.064***			
Constant		−2.08	0.73	–
Nonverbal reasoning		1.46	0.17	0.42***
Short-term memory		0.19	0.06	0.15**
Decoding		0.49	0.09	0.27***
Step 3	.30***			
Constant		−1.51	0.54	–
Nonverbal reasoning		0.85	0.13	0.24***
Short-term memory		0.14	0.05	0.11**
Decoding		0.23	0.07	0.12**
Vocabulary breadth (oral)		0.33	0.16	0.10*
Vocabulary breadth (written)		1.31	0.17	0.38***
Vocabulary depth		0.60	0.15	0.17***
Connection strength		0.45	0.13	0.13***
Total adjusted R^2^	.645			

* *p* < .05; ** *p* < .01; *** *p* < .001

### Individual effect of vocabulary measures on reading comprehension scores

To gain insight into the individual predictive quality of each semantic measure, additional analyses were conducted. To understand the predictive quality of a single predictor, two regression models have to be compared: a complete model in which all predictors are present and a reduced model in which the predictor of interest is left out. This procedure was adopted to examine the unique contribution of each lexical predictor in explaining individual differences in reading comprehension. The complete model was tested in the previous part of this results section. Four reduced models were fitted, one for each of the lexical predictors. These reduced models were compared to the complete model including all predictors in order to examine the unique contribution of each lexical predictor in explaining individual differences in reading comprehension.

The results indicated that each lexical predictor explained unique variance in predicting reading comprehension. Written vocabulary breadth explained 8 % of the variance in reading comprehension, after controlling for nonverbal reasoning, short-term memory, decoding, and the other three vocabulary measures: *F*(1,284) = 60.95, *p* < .001, ∆*R*_*adj*_^2^ = .08. In addition, vocabulary depth explained an extra 2 % of the variance in reading comprehension, after controlling for the other variables: *F*(1,283) = 15.81, *p* < .001, ∆*R*_*adj*_^2^ = .02. Furthermore, after controlling for the other variables, differences in connection strength explained an extra 1 % of variance in reading comprehension: *F*(1,284) = 12.08, *p* < .001, ∆*R*_*adj*_^2^ = .01. Finally, a small portion of the variance in reading comprehension (0.4 %) can be explained by differences in oral vocabulary breadth, after controlling for the other variables: *F*(1, 284) = 4.38, *p* = .04, ∆*R*_*adj*_^2^ = .004.

Taken together, these results indicated that reading comprehension skills were better developed in children who knew more words, had a deeper understanding of word meanings, and had stronger connections between words. Together, the four measures predicted 30 % of the variance in reading comprehension. Of this 30, 18.6 % was shared between the four indicators of semantic quality. Each measure also uniquely explained some additional variance in predicting reading comprehension. Although these effects were relatively small, all effects were (highly) significant. Of the four lexical measures, written vocabulary breadth was the strongest lexical predictor of individual differences in reading comprehension skill.

## Discussion

The general aim of the present study was to examine more closely the relationship between the mental lexicon and reading comprehension skills in fourth graders. The question we answered with the current study was: How are individual differences in the mental lexicon related to individual differences in reading comprehension skills? Two tests were used to assess the number of lexical entries (vocabulary breadth): one based on oral language and one based on written language. The quality of semantic knowledge (vocabulary depth) was measured with a definition task. Finally, the strength of connections between representations was determined with a word association task. We hypothesized that individual differences in each of these lexical aspects would be related to individual differences in reading comprehension skills. The results supported our hypotheses; all four measures explained unique variance in reading comprehension skills.

Lexical knowledge was a significant predictor of reading comprehension skills while controlling for general reasoning, short-term memory, and decoding. These results line up with previous research (e.g., Cutting & Scarborough, 2006; Ricketts et al., 2007; Verhoeven et al., 2011). Of the total 65 % of explained variance in the current study, the four vocabulary measures combined explained 30 % of the variance in reading comprehension skills. Compared to previous studies, this is a relatively large amount. Ouellette (2006), for example, concluded that vocabulary explained 15 % of the variance in reading comprehension among fourth-grade students. Our design might be the reason why, when compared to other studies, a relatively large amount of variance is explained by the vocabulary measures. Although it has been suggested that the mental lexicon plays an important role in reading comprehension (e.g., Perfetti & Stafura, 2014), the complex nature of this storage component is not often considered when examining the role of vocabulary in reading comprehension. In the present study, we used a multicomponent approach in which different aspects of the lexicon were measured. The current results imply that testing only one dimension of the lexicon might bias the interpretation of the relationship between vocabulary and reading comprehension. The size of this relationship could be underestimated when using one or a limited number of measures.

Previous research has suggested that a relationship exists between size of the lexicon, depth of lexical knowledge, and strength of connections between representations, on the one hand, and reading comprehension, on the other hand (Betjemann & Keenan, 2008; Braze et al., 2007; Hall, Greenberg, Laures-Gorse, & Pae, 2014; Landi, 2010; Ouellette & Beers, 2010; Ricketts et al., 2007; Tilstra et al., 2009; Van Steensel, Oostdam, Van Gelderen, & Van Schooten, 2014; Veenendaal, Groen, & Verhoeven, 2015). The current study, however, was one of the first to combine these different lexical aspects into one design and examine each individual contribution in explaining individual differences in reading comprehension. As hypothesized, individual differences in reading comprehension skill could be predicted by individual differences in size of the lexicon, depth of lexical knowledge, and strength of connections while controlling for decoding skills, short-term memory capacity, and general reasoning. The results from the multiple regression analyses indicated that all lexical aspects contributed uniquely in explaining individual differences in reading comprehension skills. These results are in line with the complexity and centrality of the lexicon as proposed by Perfetti and Stafura (2014). Of the four semantic quality measures, the written vocabulary breadth test explained the most unique variance in predicting fourth graders’ reading comprehension skills (8 %). According to the LQH (Perfetti & Hart, 2002) representations consists of three chunks of information (i.e., orthographic, phonological, and semantic). The task measuring written vocabulary breadth and the reading comprehension tasks rely on all three constituents: the orthographic and phonological constituents in decoding and the semantic constituent for retrieving meaning. The overlap between the two tasks might be why the written vocabulary breadth task, of the four semantic quality measures, was best predictive of reading comprehension.

Although all lexical predictors explained unique variance in predicting reading comprehension, it should be noted that almost two-thirds of the total variance in predicting reading comprehension explained by the lexical measures was shared. Based on these results, we conclude that the various lexical components measured in the present study show overlap and can be considered to be part of the same construct. However, the additional unique variance explained in reading comprehension by each individual predictor suggests that, in addition to this shared construct, each component also has something unique to offer.

The results of the current study need to be interpreted with some caution. In the current study no measure of listening comprehension was included. It has been very well established that listening comprehension is strongly related to reading comprehension (SVR; Hoover & Gough, 1990). Previous work has indicated that, in contrast to the original theory, the listening comprehension components of the SVR should be regarded as a more general linguistic component including vocabulary knowledge (e.g., Tilstra et al., 2009; Verhoeven & Van Leeuwe, 2008). Therefore, listening comprehension and vocabulary might share variance in predicting reading comprehension skills. Future research on the unique contributions of different dimensions of vocabulary in explaining differences in reading comprehension could benefit from including a measure of listening comprehension.

As suggested by previous research, reading comprehension tests differ in the underlying skills they assess (e.g., Keenan et al., 2008; Kintsch, 2012; Nation & Snowling, 1997). Therefore, in the current study, we used four different tests covering different text types, different text lengths, and different types of questions. The purpose of the current study was to examine the relative contributions of the various aspects of semantic quality to reading comprehension in general. Future research exploring whether the relative contribution of the various aspects of semantic quality to various dimensions of reading comprehension (e.g., text length and text type) would be highly interesting, and the results might have important implications for both education and research.

The combination of findings presented in this paper offer important implications. Given that lexical measures related to lexical size, lexical depth, and strength of connections each explained unique variance in reading comprehension skill, future research on the role of vocabulary in reading comprehension should consider the complex nature of this relationship and include measures of these different dimensions of vocabulary. For educational purposes, the current results indicate that difficulties in reading comprehension might originate from a variety of lexical problems. Accordingly, vocabulary instruction in education should focus not only on increasing the number of lexical entries in the lexicon, but also on depth of knowledge and on the connections between words. Enhancing all these aspects of the lexicon may lead to an increase in reading comprehension skills. However, intervention studies on these different aspects of the lexicon and their relationship to reading comprehension are warranted. Finally, in order to make causal claims, future research could benefit from adopting a longitudinal perspective in which developmental trajectories are examined.

## Conclusion

The results of the current study suggest that various aspects of lexical knowledge might be related to reading comprehension skills in different ways. Individual differences in fourth-grade reading comprehension skills can be explained by differences in the number of lexical entries (vocabulary breadth), semantic quality of these entries (vocabulary depth), and connection strength between lexical representations. In research, this complex relationship between the lexicon and reading comprehension should be taken into account. For education, vocabulary instruction should focus on enhancing the lexicon by increasing the number of representations stored in the lexicon and enhancing the quality and connection strengths of these representations.
